# Teaching students to think spatially through embodied actions: Design principles for learning environments in science, technology, engineering, and mathematics

**DOI:** 10.1186/s41235-016-0039-y

**Published:** 2017-03-20

**Authors:** D. DeSutter, M. Stieff

**Affiliations:** Department of the Learning Sciences, University of Illinois-Chicago, Chicago, IL 60607 USA

**Keywords:** Mental Rotation, Conceptual Knowledge, Spatial Ability, Spatial Concept, Spatial Thinking

## Abstract

Spatial thinking is a vital component of the science, technology, engineering, and mathematics curriculum. However, to date, broad development of learning environments that target domain-specific spatial thinking is incomplete. The present article visits the problem of improving spatial thinking by first reviewing the evidence that the human mind is embodied: that cognition, memory, and knowledge representation maintain traces of sensorimotor impressions from acting and perceiving in a physical environment. In particular, we review the evidence that spatial cognition and the ways that humans perceive and conceive of space are embodied. We then propose a set of design principles to aid researchers, designers, and practitioners in creating and evaluating learning environments that align principled embodied actions to targets of spatial thinking in science, technology, engineering, and mathematics.

## Significance

This work brings what we know about learning with the body to the design of learning environments for spatial thinking. Spatial thinking is important at all levels of science, technology, engineering, and mathematics (STEM) and students who struggle to understand the relationship between space and their chosen discipline are less likely to pursue STEM careers. The article addresses the critical issue of curriculum reform in STEM by synthesizing theoretical and empirical work across psychology, neuroscience, and the learning sciences to propose principles for design that can guide efforts to produce novel curricula.

## Background

Spatial thinking is a central component of the science, technology, engineering, and mathematics (STEM) curriculum (Newcombe, 2010). In beginning and advanced classrooms, students must reason about how spatial relationships and their changes over time impact physical and biological systems. This type of thinking can range in complexity from comparably simple (e.g., computing the distance between two points in Cartesian space) to substantially complex (e.g., predicting the way that large macromolecules such as enzymes fold in order to interact with other biomolecules). Despite the pervasive need to think spatially across the curriculum, spatial thinking is still largely an epiphenomenon of instruction. The lack of direct attention to spatial thinking during teaching and learning is problematic, given that the capacity to reason accurately about spatial relationships and their changes over time has been cited as a primary barrier to success in STEM classrooms and to entry in STEM careers (Uttal et al., 2013). Recent research in psychology, neuroscience, and the learning sciences suggests that spatial thinking may be improved through instruction that has students engage in spatial concepts and ways of thinking with their bodies. Such instruction may be particularly potent toward improving spatial thinking given an increasing corpus of evidence that human knowledge about space is embodied.

Preparing the next generation of STEM professionals requires critically examining and re-envisioning teaching and learning settings so that spatial thinking is elevated to a targeted objective of learning and is engendered as an essential habit of mind (National Research Council, 2006). First and foremost, spatial thinking is exercised extensively by experts: scientists across the STEM spectrum regularly reason about the relationship between the concrete and theoretical entities they study, their complex spatio-dynamic properties, and how these properties give rise to macroscopic observations. The ability to think spatially has led to some highly visible and significant scientific breakthroughs (National Research Council, 2006; Uttal & Cohen, 2012): Watson and Crick’s construction of the DNA double helix, Rutherford’s deduction of the presence of the nucleus, and Pasteur’s account of light polarizing tartaric salts; all represent discoveries that required extensive thought about the relationship between space and the physical properties of nature. Moreover, retaining and producing more successful graduates in STEM disciplines represents an economic and strategic imperative shared across OECD member countries (OECD, 2012). In the USA, the Obama administration recently set a goal of producing 1,000,000 new STEM graduates within the next decade. Troublingly, of the students enrolling in STEM degree programs, currently only 40% are following these degrees to completion. Students leave the STEM pipeline for myriad reasons, but challenges related to spatial thinking have been identified as an important factor that contributes to their pursuit of alternative careers (National Research Council, 2006; Uttal et al., 2013).

The call for reform is clear and many learning environments have emerged in which students are trained in the critical aspects of spatial thinking. These learning environments, which are physical and cultural contexts engineered with the express purpose of facilitating the attainment of target learning objectives by a specific audience, vary vastly in their design principles. Where some attempt to improve STEM outcomes by training students in domain-general skills (Miller & Halpern, 2013; Sorby, 2009), others leverage domain-specific tools, such as molecular models, to help students reason about complex spatiotemporal dynamics (Stull, Hegarty, Dixon, & Stieff, 2012). However, despite such efforts to give students training in spatial thinking to improve outcomes in the STEM classroom, these learning environments make different assumptions about the nature of spatial thinking and, as such, propose substantially different solutions to support students as they learn to reason about space. Given the variation in assumptions in the literature, a fundamental question needs to be addressed: what exactly do we mean by *spatial thinking*?

### On defining spatial thinking and its importance in the curriculum

Researchers have historically focused on the relatively narrow cognitive construct of spatial ability to quantify human propensity to reason about space. Studies of spatial ability grew out of a long history of psychometric development in early psychology and this line of work assumed that humans have some genetically determined baseline capacity to perceive, mentally visualize, and manipulate spatial relationships. Research in this tradition has argued that individual and group differences in spatial ability are a prominent causal factor that can account for attrition in the STEM pipeline. This position argues that students with low spatial ability are less able to visualize and operate on the spatial relationships that underlie disciplinary concepts in STEM and that this negatively impacts these students very early in their course of study (Uttal & Cohen, 2012; Wai, Lubinski, & Benbow, 2009). A number of correlational studies have attempted to establish the link between spatial ability and spatial thinking in STEM by demonstrating correlations between spatial ability and course-based achievement measures in physics (Kozhevnikov, Motes, & Hegarty, 2007), chemistry (Bodner & McMillan, 1986; Pribyl & Bodner, 1987), and geology (Kali & Orion, 1996) among many others (for a review cf. Hegarty, 2014). These findings have been further bolstered by longitudinal analyses that show persistent relationships between measures of spatial ability (e.g., mental rotation, paper folding, and perspective taking) and the likelihood an individual will perform well in STEM courses and pursue a career path in STEM (Wai et al., 2009).

The logic goes that if individual differences in spatial ability are in fact an important causal factor that cascades through a student’s immediate success and later career choice, students of lower spatial ability are selectively disadvantaged by current curricula and should be supported through training targeted at improving individual spatial ability. Recent meta-analytic work has provided further impetus for a focus on training spatial ability, concluding that spatial ability is in fact responsive to instruction (Uttal et al., 2013). Some notable studies have even empirically demonstrated that training students in domain-general spatial tasks positively correlates with higher achievement scores on domain-specific outcome measures (Small & Morton, 1983; Sorby, 2009). However, despite the empirical rationale to design learning environments that teach domain-general spatial ability as a means to improve STEM achievement, critiques against this line of work argue that correlations between spatial ability and achievement remain weak to moderate (*r* = 0.2 to 0.3; Hegarty, 2014). Moreover, extant work has not clearly established that the relationship between training spatial ability and STEM achievement is causal (Stieff & Uttal, 2015). Even in studies where spatial ability is improved through sustained practice, the benefits of the training do not transfer unilaterally across all STEM disciplines and these benefits decline rapidly after training (e.g., Miller & Halpern, 2013).

While traditional conceptions of spatial ability are clearly related to STEM career choice and course achievement, measures of spatial ability may simply not capture the more complex forms of spatial thinking that characterize STEM curricula and the disciplines more broadly (National Research Council, 2006). In fact, although spatial ability is an independent predictor of performance and attainment in STEM, this relationship does not hold for experts and may not characterize the rich knowledge base that experts draw on (Uttal & Cohen, 2012). Hegarty (2014) argued that the lack of clear causal evidence linking increases on measures of spatial ability resulting from training to improved performance in STEM disciplines opens the question of how spatial skills should be taught: should the focus be on domain-general skills such as mental rotation or do training studies need to be designed around “… the context of specific [disciplinary] content (e.g., facility in rotating molecules in chemistry)” (p. 151).

A broader interpretation of the cognitive components that learners require to succeed as spatial thinkers may be generative toward identifying the shared and domain-specific challenges in the STEM curriculum. Such work may also better identify instructional targets that can mitigate the challenges learners have thinking spatially that are not captured in domain-independent training regimes. *Spatial thinking* is a more generalized construct that “…is broader than spatial ability and related concepts in that it approaches the process of problem solving via the coordinated use of space, representation, and reasoning” (National Research Council, 2006, p. 27). Spatial thinking in this sense acknowledges that concepts of space are often drawn on by multiple disciplines, but that the *application* of spatial concepts is entwined with discipline-specific practices. For example, although engineers and mathematicians both reason about the *extent of objects in space*, engineers might do so in the context of a computer-assisted design (CAD) application where they design precisely balanced machine parts that they dynamically view from multiple angles. A mathematician, on the other hand, may use a numerical computation engine such as MATLAB to investigate optimization procedures for hypersphere packing in non-Euclidean *N*-dimensional hyperbolic space.

While these examples both draw on the same concept of space (“extent of an object”), the notion of spatial thinking provided by the National Research Council (2006) highlights that the contexts of use and forms of representation used by an engineer and a mathematician as well as the rationale propelling their lines of work differ in important ways. Because the spatial thinking construct brings a focus to the use of space during problem solving, it readily subsumes many traditional conceptions of visuospatial ability, but it also meaningfully extends the analysis of problem solving to consider the representational contexts of use (e.g., diagrams, maps, computer visualizations) as well as the processes of reasoning (e.g., drawing on concepts of space to structure a problem solution and construct inferences about a possible solution strategy). The power of the spatial thinking construct rests precisely in its ability to focus attention on shared aspects of spatial thought across STEM while also highlighting that these shared practices instantiate differently depending on the disciplinary context. There is no short list of concepts that are expressed in a language of space: biologists use space to explain the structure–function relationships in biological systems, chemists use space to explain how molecules are structured and interact during a reaction, civil engineers use space to design skyscrapers and test the impacts of wind shear. Each discipline reasons about space, but employs different ways of expressing space via representations and tools, and employs specific ways of framing disciplinary problems and scientific inquiry.

Reframing spatial thinking in this way will not only be generative toward conceiving new learning environments that exist outside the historically narrow notions of how to improve STEM achievement, it also has the potential to address misconceptions around who can succeed in science. This characterization of spatial thinking does not mandate a singular approach to curriculum reform: rather, it suggests that diverse learning environments can address spatial thinking in a discipline in substantially different ways. This flexibility provides ample creative space for researchers and practitioners to consider ways to increase students’ “STEM dose” early on and potentially increase the likelihood that they pursue STEM careers in their professional lives (cf. Wai, Lubinski, Benbow, & Steiger, 2010). More crucially, conceiving of spatial thinking as a flexible framework with no singular application affords significant intellectual diversity: research questions can move away from framing learning in deficit, as in the case of training spatial ability. This is particularly consequential when considering that the targets of these deficit models often and disproportionately are women and historically underrepresented minorities (Newcombe & Stieff, 2012).

In our research, we study how spatial thinking can be improved by designing learning environments that have students use their bodies to represent and enact spatial concepts *vis-à-vis* discipline-specific representations and processes of reasoning. From infancy, humans interact with the world through multiple senses. Babies use their hands and feet to push on their surroundings and receive direct perceptual feedback from their eyes, ears, and sense of touch about the consequences of their actions. The closely coupled feedback between actions and perception shapes the way in which humans begin to form internal representations of their world (Wellsby & Pexman, 2014). In fact, before an infant is even able to talk, it develops a basic sense of physical cause and effect: if a block has been pushed off a ledge, infants are puzzled if the block does not then immediately fall. This process of generating mental representations with our bodies extends well beyond infancy. When children reason about the concept of number and the operations of addition and subtraction, they often use their fingers to represent these abstract ideas. Furthermore, research in the field of gesture for learning supports the idea that having students use their hands and bodies while learning complex concepts improves their memory and reasoning about those concepts. For example, students taught via gesture about the concept of the slope of a line better understand the ideas of “increasing and decreasing” trends (Alibali et al., 2013). The process by which physical action in the world generates, stores, and reactivates mental representations abstracted from bodily experience is what is often referred to as *embodiment*.

## Embodied cognition and spatial thinking

Although there is as yet no unified theory of embodiment, scholars of embodied cognition generally agree that mental processes are mediated by body-based systems, including body shape, movement, and scale; motor systems, including the neural systems engaged in action planning; and the systems involved in sensation and perception (Alibali & Nathan, 2012, p. 248).

### The embodied mind

Theories of embodied cognition propose to explain the genesis of human conceptual knowledge representation and cognitive processing as rooted to varying degrees in the shape of the human body and its action with the environment. The pursuit to unify various aspects of cognition with respect to bodily form and action, however, has given rise to various interpretations of the “embodiment” construct and the degree to which behavior and knowledge representation can be viewed as body-based. Given the polysemous nature of embodied cognition and an incomplete theoretical parsimony in the literature, we first clarify what precisely we mean by embodiment, the evidence taken to support this account, and what implications should arise from this theoretical framing.

Although theories of embodied cognition have been around for decades, there is no singular view of what is meant by the term “embodiment” or embodied action (Anderson, 2003). In a broad sense, embodiment is characterized by a shared assumption that the body, its particular form, and its sensory capacities supply a cognitive system with a rich input stream that shapes knowledge representation and later cognitive processing of those representations. However, despite this class of shared assumptions, researchers operationalize “the embodied mind” differently in empirical work and it is not always clear that embodiment is an umbrella term for lines of work separated by differing philosophical assumptions. Wilson (2002) has provided a succinct review of these threads, their assumptions, and the claims they have advanced. Embodiment, as referenced in this article, is consistent with Wilson’s sixth claim: namely, that *offline cognition is body-based*. Embodiment in this sense is a fundamentally brain-based phenomenon, where, “[the] function of the sensorimotor resources is to run a simulation of some aspect of the physical world, as a means of representing information or drawing inferences” (Wilson, 2002, p. 633).

Some important existence proofs of embodiment have arisen out of studies of semantics, suggesting that meaning and knowledge representation engage sensorimotor simulation. Humans are quicker to identify whether a common household tool (e.g., frying pan, hammer) is in the correct or inverted orientation when the stimulus is presented in an orientation consistent with how a human would grasp that object for action (Tucker & Ellis, 1998). In the action-compatibility effect (ACE), Glenberg and Kaschak (2002) found that participants are quicker to identify whether a phrase is sensical when participants move their body in a way congruent with the motion implied by the sentence. For example, if participants were asked to press a button that required extension of the arm away from the body when reading the sentence “Mary pulled the drawer toward herself,” it would take longer to judge this sentence as sensical than the condition where the motion and semantic meaning of the sentence were aligned.

Further behavioral evidence from language processing studies has shown that comprehension of certain grammatical constructions results from a mental simulation within the action space implied by the sentence. Kaschak and Glenberg (2000) demonstrated that meaning in language is not purely syntactic: when presented with innovative denominal verbs constructed from nouns, participants were quicker to read sentences as sensical when the affordances of the noun were consistent with the action implied in the sentence. For example, the sentence “the woman crutched the goalie the ball” would be judged as sensical over the same grammatical construction “the woman egg-shelled the goalie the ball,” because a crutch has particular affordances such as rigidity and extension that allow the woman to transfer the ball to the goalie that are not possible for the egg shell. Of importance, these effects require no common association between verb and object. Detectable differences in reading time are also found when participants read sentences that combine the affordances of objects that exhibit no typical association, but whose affordances mesh during mental simulation (e.g., “hang the coat on the vacuum”). Kaschak and Glenberg argue that a view of language comprehension as a manipulation and combination of abstract symbolic knowledge would not predict such reading time differences. Further, they argue that these studies lend evidence to support a view that humans draw on modality-specific information even though they are displaced in space from the scene, actors, objects, and syntactic relationships implied by a sentence.

Such behavioral findings are consistent with Wilson’s (2002) body-based view of offline cognition. A growing number of neuroimaging studies have also sought to directly image the brain regions engaged in these object recognition and conceptual processing tasks. For example, the motor system is shown to selectively activate when individuals observe objects that have common action affordances or read action-related words. Functional magnetic resonance imaging (fMRI) studies have demonstrated that when right-handed participants see images of tools (e.g., a hammer), higher levels of activation are observed in the left ventral premotor cortex compared to viewing objects with no typical associated hand movements (e.g., an elephant), suggesting that perception of manipulable objects automatically elicits imagined interactions with those objects (Chao & Martin, 2000). When participants read action-related words such as *kick*, *pick*, *lick*, motor-specific leg, hand, and mouth areas of the brain activate in response to the word, indicating sensorimotor activation in the comprehension of these words (Pulvermüller, 2005). Moreover, lesion simulations via transcranial magnetic stimulation (TMS) have been shown to induce differences in behavioral performance with respect to comprehension of such action words (Pulvermüller, Hauk, Nikulin, & Ilmoniemi, 2005).

These empirical findings provide substance to the claim that cognition and knowledge representation engage body-based simulation. However, note that we reject the “strong” formulation of the embodiment hypothesis given that it is incongruous with available neuroscientific evidence (Chatterjee, 2010; Meteyard *et al*., 2012). That is, the claim that human knowledge and cognitive processing are *completely* embodied and are composed *solely* of sensorimotor content is an untenable position. Recent critiques have emphasized that while the evidence clearly shows that modal areas of the brain activate during conceptual processing, it has not been ruled out that a more abstract form of conceptual knowledge does not simply cascade to these areas of the brain in a functionally unimportant manner (Mahon & Caramazza, 2008). Meteyard *et al*. (2012) reviewed a number of theories of embodiment and claims advanced about semantic processing in the face of neuroscientific and neuropsychological literature. The theories of embodiment were graded on a continuum and characterized as: (1) non-embodied, (2) secondary embodiment, (3) weakly embodied, or (4) strongly embodied. At one end of the continuum, non-embodied theories encompass traditional cognitivist views of representation. At the other end, strongly embodied theories argue that primary perceptual cortices are directly recruited in knowledge representation and that veridical sensory impressions are simulated during semantic grounding. In the middle of the continuum, theories of secondary and weak embodiment disagree primarily on the extent to which the modal regions of the brain are directly implicated in knowledge representation. Theories of secondary embodiment propose “that the semantic system is independent of but directly associated with sensory and motor information,” whereas theories of weak embodiment “propose that semantic representations are at least partly constituted by sensory-motor information” (Meteyard *et al*., 2012, pp. 791–2).

Meteyard and colleagues (2012) argued that the current evidence best aligns with claims from weak embodiment, where functional neural clusters have been found in regions parallel (e.g., anterior) to primary sensory cortices. Consistent with the functional-anatomical hypothesis (Chatterjee, 2008), weak embodiment argues that functional neural clusters that are organized near primary perceptual cortices, but non-isomorphic to them, function to abstract features of experience and provide input into higher-order representational systems. In a weakly embodied view, abstracted modal experiences converge during the access of mental representations in convergence zones, where simulation “…may instead be the activation of feature conjunctions sufficient to represent a given object, or word” (Meteyard *et al*., 2012, pp. 794–5).

Establishing that knowledge is not solely embodied does not rule out the possibility that all knowledge has *some* embodied component. Addressing this possibility in its full complexity will likely require years of targeted behavioral and brain imaging studies, but current evidence suggests that cognitive processes that recruit mental imagery appear to have a more strongly embodied character: “…we find ourselves supporting a position where primary sensory and motor regions are not activated during routine semantic processing (in opposition to strong embodiment) but may be so for deeper processing related to imagery” (Meteyard *et al*., p. 801). Despite the ongoing debate, the stance advanced by Chatterjee (2010) that an embodied/disembodied dichotomy has “outlived its usefulness” in face of the neuroscientific evidence moves the field beyond asking questions about the existence of embodiment. Instead, nuanced research questions that probe *when* and *to what extent* conceptual knowledge is embodied are likely to be more generative moving forward. Therefore, embodiment is better understood through the *grounding* metaphor: that particular concepts, and even classes of concepts, are grounded in perception and action states from an individual’s prior experience and that such conceptual knowledge is mediated by simulation as a function of task demands.

Such behavioral and brain imaging studies provide a strong counterpoint to traditional cognitivist models of mental representation and computation (e.g., Newell & Simon, 1972; Pylyshyn, 1984) where amodal symbols exhibit non-analog mappings to the external world. Instead, complementing lines of evidence support the view that important concept-driven processes (e.g., language comprehension) can draw on simulation of analog properties of the body and experience with the external world to ground meaning. Consistent with the reviewed literature, grounded views of embodiment do not position human perception as a veridical recording system (Barsalou, 2008; Meteyard *et al*., 2012). Rather, visual, auditory, kinesthetic, olfactory, and somatosensory experience provide a rich input spectrum to the cognitive system, from which features, relationships, and states are schematically abstracted — a process that is also subject to error — that can generalize beyond the immediate situation in which they were produced (Barsalou, 1999).

As theories of embodiment are translated into accounts of learning, an open question remains about the mechanism by which new knowledge – especially abstract knowledge – can be accounted for within an embodied framework and how neurally abstracted sensory impressions are implicated in broader cognitive representation. While settling on a specific mechanism is outside the scope of this paper, a few possibilities are worth considering. The first is that embodied actions may provide students with novel representations for structuring information and problem solving. In this view, the performance of actions with the body provides learners with new representations, such as representational gestures, that foreground aspects of a domain in a stable form that can be readily reproduced. These body-based representations might become part of a learner’s “toolbox,” providing utility for reasoning on novel tasks, where bodily action might serve to alter the way in which the learner structures their thinking.

A second possible mechanism is analogy/metaphor. Analogy and metaphor have long received attention as a mechanism by which sensorimotor impressions derived from experience help structure more abstract reasoning (Lakoff & Johnson, 1980, 1999; for a review see Jamrozik *et al*., 2016). In this view, individuals repeatedly access sensory and motor impressions of concrete objects and events and come to abstract the more generalizable relational properties of these instances that apply to other knowledge. For instance, the phrase “negotiation is a muscle” could be understood by first accessing sensory impressions of muscles: muscles are flexible and can apply force; muscles can be strengthened with practice. These generalized relationships can then become ascribed to “negotiation” in a way that is not made explicit in the turn of phrase alone. Of importance, fMRI work has constrained this view: sensorimotor simulation both varies as a function of the experiential nature of the source of the metaphor as well as the actual accrued exposure the individual has perceiving and interacting with the source of that metaphor (Jamrozik *et al*., 2016). Such a mechanism is wide-ranging in its explanatory capacity and resembles other accounts, such as Barsalou’s (1999) *perceptual symbol systems,* where “multimodal traces of neural activity that contain at least some of the motor information present during actual sensorimotor experience” (Goldin-Meadow & Beilock, 2010, p. 665) can ground meaning in simulation.

A third possible mechanism is that performing actions with the body may serve to sharpen existing spatial representations that a learner may already have access to. In spatial domains, the analogy/metaphor mechanism brings into question what precisely is the source from which source-target mapping may proceed. This would imply that to think spatially may actually involve the reactivation of an individual’s prior representation of a spatial concept and make it salient in the new context. This existing spatial representation, perhaps in the form of a motor or image schema, can then be mapped to the novel task in a way that may hone the existing representation.

This integrative view of mind and body is a departure point from which to reframe what is at the disposal of a learner in a learning environment and what potential consequential utility might result from instruction that aims to promote embodiment around STEM concepts. Given the previous assertion that the “strong” form of embodiment is untenable, it is also not supported by current evidence that *all* facets of knowledge accessed during problem solving in STEM tasks merely lack some embodied alternative. However, there is evidence in extant literature that human concepts of motion in space and the representation of spatial relationships is a class of knowledge that frequently recruits modal simulation and that the body should thus serve as an inroads to promoting domain-relevant understanding of spatial concepts.

### On the embodiment of spatial thought

Embodiment is not novel per se in investigations of human spatial thought and cognition. Developmental psychologists have long explored the connection between body–environment feedback, concept formations, and development of mental representations (cf. Wellsby & Pexman, 2014). Piaget’s observations of his children and their development of push/pull schema from interacting with blocks progressively removed from their immediate reach showed how infants learn about the allowed classes of interactions with their environments through action and perception feedback (Piaget, 1952; Piaget & Inhelder, 1956). In robotics and artificial intelligence, researchers found that by providing robots with biological perceptuomotor systems able to perceive, process, and encode aspects of the external world they could create a form of intelligence that emerged in the absence of rich explicit internal representations of the environment (Brooks, 1991; Kirsh, 1991).

Recently, Waller (2014) argued that “[s]patial thought may be an excellent venue for [the modal basis of internal spatial representations], and may be relatively better poised than many other research domains to provide evidence for the constitutive claims of embodied cognition” (p. 148). Many tasks that are used as proxy measures for the human capacity to decode and manipulate spatial entities rely on analog mental simulation. For example, Shepard and Metzler’s (1971) canonical finding of a linear relationship between an individual’s response time and the angular disparity between two block pairs in tests of speeded rotation suggests that processes like mental rotation rely on imagistic manipulation of the blocks as if they were actually being moved in the physical world. Moreover, interference studies have demonstrated that on the block rotations of Shepard and Metzler, when individuals are asked to rotate a joystick either aligned or anti-aligned to the required mental manipulation of the blocks, detectable response time differences emerge where the aligned condition is quicker (Wexler, Kosslyn, & Berthoz, 1998). Chu and Kita (2008, 2011) have shown that individuals who gesture to solve similar mental rotation tasks outperform their non-gesturing counterparts and that this is a trainable skill.

If spatial knowledge exhibited a non-analog correspondence to modal experience, then preferred reference frames in spatial memory tasks should also be an unobserved phenomenon (Waller, 2014). In tasks probing judgment of relative distance, participants consistently prefer to encode the location of objects with respect to their natural corporeal orientation to gravity. Rather than observing a chance distribution of a participant’s reference frame with respect to an array of objects, there is a strong bias to orient the upward *z*-axis with the viewer’s bodily axis. In addition to preferring the vertical bodily axis, Franklin and Tversky (1990) have also demonstrated that location judgments are not made equally along all bodily axes. Testing the *spatial framework hypothesis*, Franklin and Tversky showed that an isotropic notion of space is undercut by response time biases where individuals in an upright position were fastest to identify objects from an imagined array above and below them, slower to identify objects in front of or behind them, and slowest to identify the location of objects on a lateral left–right body axis. Moreover, Kosslyn, Ball, and Reiser (1978) observed that spatial representations in memory preserve metric properties. Kosslyn and colleagues demonstrated that irrespective of whether a participant viewed an actual spatial pictorial representation or simply imagined one, response times on tasks where individuals were asked to scan the image were nearly identical. Taken together, these findings point to an understanding of space that is modal and analog.

Some of the earliest empirical evidence supporting embodiment of spatial thought in modern psychology originated in cognitive linguistics. By investigating the implicit conceptual structure embedded in human language, Lakoff and Johnson (1980, 1999) argued that human concepts are fundamentally grounded in bodily experience and arise from experience with the world. In particular, Lakoff and Johnson provided evidence that spatial concepts are fundamentally embodied. They cited cross-linguistic analyses of spatial language to show that despite millennia of separate evolution, various human languages contain remarkably similar concepts of space that map to the anatomy of the human body. In English, for example, the constructions “the ball is on top of the box” or “the dog is behind the tree” can be interpreted from an egocentric reference frame that conceptualizes spatial relationships structurally isomorphic to the human body. That is, in the first sentence “top” designates the point on an object most distal from the pull of gravity. For a human this location corresponds to the head and, by extension, the same location on the box. In the second example, the tree acquires the attributes “front” and “back” because one hemisphere of the tree is in view (as would be the case in human discourse) while the other hemisphere is occluded from vision.

The use of the body and embodied knowledge to represent and think spatially has also been identified among expert STEM professionals engaged in their discipline. Ethnographic studies of scientists engaged in authentic practice have found that complex spatial ideas are often conveyed using representational gesture-based and body-based metaphors. When explaining the complex configuration of the protein thrombin, research biologists frequently recruited representational gestures to demonstrate the complex conformational changes of the protein in the presence of thrombomodulin with their hands (Becvar, Hollan, & Hutchins, 2005). In a study of physicists collaboratively working to understand the relationship between temperature and magnetic transitions in a particular material, Ochs, Gonzales, and Jacoby (1996) found that scientists drew on body-based metaphors during discourse when they were confused about novel hypotheses (“When I come down I’m in the domain state”). Moreover, Nobel Laureate geneticist Barbara McClintock has long been recognized for her innovative approach to imagining herself as the plants she studied, “perceiving” the chromosomes (Henriksen, Good, & Mishra, 2015). McClintock’s work lead to a number of significant breakthroughs in scientific understanding of gene expression, exchange of genetic information during meiosis, and preservation of information in telomeres and centromeres.

Neuroimaging studies have only recently begun to probe how the brain represents spatial information and the connection between spatial perception, conception, and language. When individuals view a visual scene and are then asked to imagine it in the absence of the stimulus, as many as 90% of the voxels that are active during online perception are also activated during imagined viewing of the scene (Ganis, Thompson, & Kosslyn, 2004; Kosslyn, Thompson, & Alpert, 1997). Studies of spatial language have found that the separate grammatical constructions for manner and path found in spoken language comport with the neural divergence of manner and path information along ventral and dorsal pathways. Regions of the laterotemporal cortex associated with action perception appear to mediate semantic grounding of spatial language, where more metaphoric uses of spatial language are mediated more anteriorly along the middle temporal gyrus (Chatterjee, 2010). These findings are broadly consistent with the thesis that humans conceive of space in a manner consistent with the grounded account of embodiment: mental representation maintains analog and metric properties, spatial computation interfaces with the motor system, humans exhibit a strong preference to encode spatial relationships consistent with body orientation, highly similar brain regions activate during perception and imagination of a visual scene, and spatial language reflects real regional specializations for conceiving and perceiving spatial relationships such as object manner and path. Thus, we propose that if *conceptual knowledge of space is mediated by body-based systems, conceptual knowledge of space should be groundable through bodily action*.

### Promoting spatial thinking through embodied actions

National reform efforts have emphasized that fostering spatial ways of thinking and problem solving are not broadly represented in contemporary curricula (National Research Council, 2006). Unlike the long standing history to reform mathematics and verbal literacy education, researchers and educators have paid comparatively less attention to supporting learners at all levels to master knowledge of space, spatial concepts, and the concomitant habits of mind that produce critical thinkers in STEM. The report argues that such a constellation of skills constitutes a particular form of literacy, subject to normative forces, where the literate student should be able to: (1) “have the habit of mind of thinking spatially,” that is, know the contexts in which it is appropriate to think spatially, (2) “practice spatial thinking in an informed way,” that is, do so in a principled manner built on a solid understanding of underlying concepts and tools of representation, and (3) “adopt a critical stance to spatial thinking”, that is, critically evaluate sources of data as well as products of problem solving (National Research Council, 2006, p. 20).

Of the aspects of spatial literacy that the National Research Council (2006) report highlights, supporting learners during problem solving to know *how to use concepts and representations of space to structure problems in a domain* is most clearly related to traditional learning outcomes. Teaching students to think spatially, then, requires considering how embodied actions can promote and “… [nurture] the practical, emotional, or *imaginative* states that are thought to undergird formal analytical thought” (Waller, 2014, p. 147, emphasis added). Embodied actions, as the name suggests, are related to embodiment (and in fact, deeply so), but the distinction between the two is important. We operationalize embodied actions as the *purposeful body positions and movements that an individual engages in during a learning activity*, where these body states and actions exhibit a non-trivial relationship to the targeted learning objective. In the case of spatial thinking, an embodied action would represent any purposeful body state or motion that reproduces a structural mapping to a spatial concept during learning. This distinction between *embodiment* and *embodied action* is important because if embodiment is ultimately a brain-based phenomenon, then embodied actions represent the physical antecedents of the embodied mind. Moreover, learning environments can only directly manipulate the physical *actions* of the learner in pursuit of promoting embodiment around a learning objective.

With this distinction in mind, there is already promising evidence that embodied actions play an important role in spatial thinking. Research in gesture for learning has shown that gesture can influence how learners approach spatial problems. For example, when students are given classic gear chaining problems in engineering, learners taught to physically trace the direction of gears are more likely to use gesture in their solution strategy (Alibali, Spencer, Knox, & Kita, 2011). Goldin-Meadow, Cook, and Mitchell (2009) showed that when students are taught to group addends with a gesture not explicitly mentioned in speech, students took up the grouping gesture as part of their explanation. Of importance, these gestures do not just influence strategy choice, they can also improve learning outcomes for students over traditional instruction. For example, students who used gestures to represent the grouping of addends in a mathematical equality were significantly more likely to mention grouping as a strategy in their verbal explanation and their gesture frequency was associated with higher scores on an achievement assessment. Similarly, chemistry students who are taught to use their hands to represent and maintain complex spatial relationships in organic molecules outperform their peers when asked to draw structural diagrams of a molecule from multiple perspectives (Stieff, Lira, & Scopelitis, 2016).

All of these examples of disciplinary learning show that gesture, and arguably embodied actions more broadly, may serve as a useful resource in improving spatial thinking precisely because it serves to ground understanding of spatial concepts in bodily action. In fact, as a manifestation of the embodied mind (Alibali, 2005; Hostetter & Alibali, 2008), gesture has been argued to enhance thought by foregrounding action in mental representation (Goldin-Meadow & Beilock, 2010). As argued previously, spatial thought may regularly be grounded in the simulation of perception and action states as a means to structure information and draw inferences: mental representations of space maintain analog features of the external world (Kosslyn et al., 1978) and mental imagistic processes such as mental rotation interface with the body’s motor system (Chu & Kita, 2008; Waller, 2014; Wexler et al., 1998). Compellingly, the imagery and computation underlying spatial thinking can be selectively enhanced through instruction employing embodied actions. For example, when presented with the classic “radiation problem” (Gick & Holyoak, 1980) that involves deciding how to irradiate a tumor without killing a patient, Craig, Nersessian, and Catrambone (2002) demonstrated that participants who were asked to make gestures that described their solution strategy performed much better than participants who only made a drawing of their solution strategy. Studies such as these demonstrate that gesture may have a unique role for supporting spatial thinking that goes beyond drawing a learner’s attention to spatial information or making spatial information more salient.

Instructionally supporting broader habits of mind to think spatially and engage in spatial processes of reasoning may also be enhanced by embodied actions. Gestures, for example, have been shown to play an important role in learning interactions: they externalize imagistic aspects of thought and coordinate shared attention around representational tools during learning (Alibali & Nathan, 2012) and speech-gesture discordance indicates a learner’s receptiveness to instruction (Alibali & Goldin-Meadow, 1993; Breckinridge-Church & Goldin-Meadow, 1986). Gestures can also feedback into and lay the foundation for new knowledge (Goldin-Meadow et al., 2009). Such research suggests that gesture has clear utility as a visible formative assessment tool, but that it also serves a broader purpose in individual cognition. Supporting learners to think spatially means considering learning environment designs that provide the imaginative mental states about concepts of space, tools of representation, and processes of reasoning that underlie more formal thought (Waller, 2014). The reciprocal quality of gesture and other embodied actions to both externalize thought as well as provide novel and retrievable resources when learning and problem solving make it a promising means through which to promote complex analytical reasoning such as spatial thinking. The position that embodied actions can act as resources to improve spatial thinking in STEM is in sharp contrast to interventionist approaches that aim to improve spatial thinking in STEM through proxy training of spatial ability.

### Constraining the breadth of embodiment

We have remained largely optimistic about the potential for embodied actions to improve spatial thinking, but we wish to constrain this position meaningfully: foremost, we strongly adhere to the philosophy that learning environments should always serve as a testbed where theories of learning can be placed in harm’s way (Cobb, Confrey, DiSessa, Lehrer, & Schauble, 2003). This means that although we are arguing that spatial thinking can be grounded in body-based simulation, we equally advocate a healthy skepticism throughout. By testing the viability of learning environments derived from theory, we can provide real evidence about whether viewing cognition as offline body-based simulation grounded in sensorimotor experience is consequential for supporting students across the STEM spectrum. Embodiment is not new to education research and various scholars have brought a unique focus to how we understand embodiment as an individual and group phenomenon. Arguably, multiple lines of investigation are beneficial if the research field is to achieve any kind of theoretical parsimony. However, despite the benefits we stand to gain from studying embodiment as it plays out *in situ*, we also argue that our knowledge of *how* and *when* embodied actions best support spatial thinking are still poorly understood and it is necessary to first investigate these mechanisms through appropriate reductionism (cf. Núñez, 2012).

We acknowledge that learning often happens through complex interactions embedded in a sociomaterial environment. Some work has even specifically sought to understand embodiment of disciplinary knowledge in social settings (e.g., Alibali & Nathan, 2012) as well as advocate distributed embodiment in supporting students’ understanding of complex systems dynamics (e.g., Lindgren & Johnson-Glenberg, 2013). While there is strength in understanding embodiment as it arises in social contexts, there are a few unavoidable confounds such an approach encounters when trying to better understand embodiment as a *cognitive* phenomenon and its role in learning. Consider a dyad working together toward understanding the concept of the water cycle. In the process, these individuals produce a rich exchange of dialogue as well as various behaviors such as posture shifts, gestures, and inscriptions to explain how water moves from reservoirs, into the atmosphere, and then back down through precipitation. Let us say that one student is explaining how “water evaporates to become clouds (*hand moves up, palm open*).” This student's partner asks a clarifying question about evaporation and performs a similar gesture. Does the second student gesture because they are simulating evaporation in a way analogous to the first student? Or, on the other hand, does this gesture now serve as a shared representation to facilitate communication? While such externalizations may provide insight into evolving spatial thinking, it is not clear whether the observed utterances result from the internal simulations that would have also been observed from each individual uniquely. Rather it is likely that the functional role of instances of language, gesture, and inscription shift fluidly for these learners as they move between individual reflection, self-explanation, attending to their interlocutor, and working to construct inferences in their dyad. Gesture, in particular, which provides insight into the non-linguistic imagistic aspects of speech, is highly susceptible to social settings: the threshold above which someone may gesture is influenced by implicit mores of the interaction (McNeill, 1992).

Such confounds to studying embodiment as a group process significantly obfuscate the ability of research to draw rigorous claims about the extent to which grounded simulation may support targets such as spatial thinking. This is especially problematic given that recent critiques from neuroscience indicate that conceptual grounding may exhibit a developmental arc. Rather than conceptual grounding being binary, it may actually evolve along a continuum toward abstract knowledge representation (Chatterjee, 2010). For example, an individual may understand the spatial concept “above” over the course of multiple exposures to arrays of objects arranged in an invariant configuration that shapes their simulation of *aboveness*. This schematization of the relationships encoded in “above” no longer pertain to any specific objects and the concept of “above” becomes flexible with respect to its referents. Chatterjee refers to this as *referential ambiguity* and argues that such abstraction is fundamental for concepts such as spatial relationships and configurations. If reasoning about space depended on first indexing specific objects, it would hold little utility in novel situations.

In addition to embodiment being subject to a developmental trajectory, spatial thinking is also a complex construct and represents no unified process (National Research Council, 2006). Spatial thinking subsumes various cognitive operations of visualizing and operating on spatial information, but it also more broadly implicates the tools and processes of reasoning that situate such cognitive competencies. We might instead assume that the role that embodiment plays in supporting learners as they develop an understanding of concepts of space, the use of tools to represent data and spatial phenomena, and the patterns of reasoning in their domain will draw differentially on simulation. In fact, the demands of some spatial tasks may more readily activate offline simulation of perceptual and motor states (e.g., imagining an object moving through space, imagining positioning oneself at different vantage points around an object) than others (e.g., employing the formalisms of the Cartesian plane to characterize the velocity of an object). Given such unanswered questions, there are likely to be rich contributions to the literature as well as theory by focusing on embodiment as a cognitive phenomenon at the individual level. The development of spatial thinking and the extent to which domain-relevant spatial concepts and processes of reasoning can be fostered through embodied action remain underexplored and warrant investigations in parallel to efforts that focus on the social nature of embodiment.

There are also likely to be individual differences with respect to embodiment that remain underexamined in extant literature. Perceptual symbols and simulators (Barsalou, 1999) represent broad theoretical constructs intended to accommodate all aspects of cognitive representation. As such, we should expect variation in the extent to which conceptual knowledge may reliably produce similar sensorimotor activation across individuals. It is comparatively easier to hypothesize that the representation of tangible objects such as tools manipulable by hand (e.g., a hammer) should more readily give rise to similar neural activation across individuals on networks associated with hand movement and action planning compared to concrete objects that cannot be moved by hand (e.g., a building). However, such a priori specificity of simulation becomes increasingly difficult as a concept becomes increasingly abstract (e.g., consider “the basis of freedom” or “world peace”). Such complexity may be attributable to Barsalou’s (1999) argument that perceptual simulators are simultaneously *rational* and *empirical.* He argues that simulators are rational because they are rooted in genetic factors. In fact, a vast majority of humans exhibit a shared anatomy and physiology – bodily and neurally – that privileges particular classes of data from the world (audition, vision, proprioception, olfaction, etc.) and is also constrained by anatomical factors of the body such as the location of important sensory organs in the head, the bilateral symmetry of the body, and the typical preferences of handedness. Simulators, though, are also empirical because humans constantly abstract and accrete sensorimotor impressions from the external world in ways that reflect idiosyncratic experience. Humans broadly share a genetic basis for simulation, but the reality that humans each inhabit a somewhat unique environment should give rise to variation in the perceptions and conceptions of the world that subserve offline cognition.

Under the notion of variable embodiment (Barsalou, 1999), individual differences should thus arise from idiosyncrasies that impact either the rational or empirical basis of simulation. Factors that would affect the rational basis of simulation arise from atypical anatomies and physiologies. For example, an individual who lacks input from some perceptual organ (e.g., deaf, blind), has a congenital defect, has had a lesion or stroke, or is born with atypical anatomy (e.g., missing a limb) has a significantly altered rational input into the cognitive system and this should give rise to unique embodiment. Within populations that do not have physiological disparities, differential embodiment should thus arise from the unique empirical experiences underlying simulation. Differences in experience occur constantly, given that no two humans inhabit the same corner of an ecological niche. In an fMRI study, ballet and capoeira dancers were both shown videos of their particular dance style as well as the style they had not had experience performing. Calvo-Merino, Glaser, Grezes, Passingham, and Haggard (2005) found that even though both groups of dancers had extensive experience engaging in their respective forms of dance, brain areas associated with action control activated more when dancers viewed a video depicting the form of dance they had personally performed. This effect was found even within a particular style of dance when male ballet dancers watched video of moves only performed by their female counterparts (Calvo-Merino, Grèzes, Glaser, Passingham, & Haggard, 2006). Thus, representation and simulation differ measurably as a function of direct experience and direct experience observing action differs from performing the action physically. In the case of the dancers, the specificity of motor movement mattered and the findings suggested that “[h]aving produced an action affected the ways the dancers perceived the action, suggesting that the systems involved in action production subserve action perception” (Goldin-Meadow & Beilock, 2010, p. 666).

## Design of learning environments for spatial thinking

The lines of evidence discussed above demonstrate that individuals can make use of embodied actions when engaged in spatial thinking and, importantly, that such actions can be trained to yield improvements in both spatial learning in a discipline (e.g., Goldin-Meadow et al., 2009; Stieff et al., 2016) and spatial abilities such as mental rotation (e.g., Chu & Kita, 2008, 2011). Of importance, these prior studies have demonstrated the effectiveness of embodied actions, specifically gestures, whether they are spontaneously produced by the learner or externally directed by someone else. These varied roles for embodied actions in spatial thinking suggest that new learning environments that wish to improve spatial learning in general or in the content of a STEM discipline should integrate embodied actions as a primary target of intervention. A common feature of embodied actions is that they are purposeful and intentional; all movement is not good movement. Embodied learning environments must aim to help learners make explicit connections between the form of a gesture or a specific action and the spatial information that is represented by the body or the action. While gestures are easily used in this way to complement verbal communication (McNeill, 1992), linking them to spatial concepts, forms of representation, or processes of reasoning that are unfamiliar or highly abstracted from everyday experience is nontrivial. As such, we propose here a set of preliminary design principles motivated by the research on embodied action that we hope will help designers develop new interventions that not only improve spatial thinking and student learning outcomes but also provide a novel context for continued study on the nature of spatial thinking and the affordances of embodied action for learning.

### Design principle #1: Embodied learning environments should include scaffolds that explicitly map spatial entities and their relationships to the hands or the body

Studies that have demonstrated the efficacy of embodied actions, specifically gestures, for promoting learning in different contexts share common features with respect to the embodied actions employed by teachers and learners. Namely, the gestures observed in these diverse studies have all been explicitly linked to discrete entities in the problem space. For example, learners have been seen to benefit from gestures that represent the combination of addends to predict a sum (Goldin-Meadow et al., 2009) or gestures that represent imperceptible phenomena such as proteins (Becvar et al., 2005). The efficacious use of gesture in this way suggests that new learning environments should help learners make clear and immediate connections between embodied actions and spatial entities to best support spatial learning. Such scaffolds must not only ensure that the embodied action is congruent with the concept to be learned (see Lindgren & Johnson-Glenberg, 2013), they must also ensure the learner can perceive the relevant congruency. For example, a gesture that represents the distance between two points on a surface or the three-dimensional structure of a molecule may promote learning about important spatial relationships in a STEM discipline, but only to the extent that the learner perceives the relationship between the gesture and the represented phenomenon. To perform the gesture without knowledge of its relevance to a learning objective is unlikely to benefit learning.

Although the design principle seems obvious, it is not the case that these relationships are immediately evident to the learner without careful scaffolding in the learning environment. In fact, several empirical studies suggest that without explicit scaffolds on the relevance of gesture for supporting learning the benefits of gesture are easily missed. For example, Howison *et al*. (2011) demonstrated that young learners can use gesture to improve their understanding of proportionality, but this occurs through a gradual process that requires direct feedback on the quality of certain gestures for representing a specific ratio. More compelling, Walkington et al. (2014) showed that adult mathematics learners also benefit from using trained gestures while learning about proofs; however, these benefits were only observed when accompanied by additional instructional scaffolds that explained to the learner how the gestures were related to underlying mathematical concepts. The critical role of instructional scaffolds that explain the relevance of gestures has also been seen in the domain of chemistry. Stieff et al. (2016) demonstrated that learners can benefit from performing gestures while learning about molecular structures only in cases where the instructor provides explicit guidance about how the hands can be configured to represent spatial relationships depicted in chemistry diagrams; when this guidance was absent, they observed no benefit of gestures even when participants performed them.

### Design principle #2: Embodied learning environments should leverage motoric actions to simulate high-fidelity spatial operations that would otherwise be imagined by the learner

Beyond representing spatial relationships within and between unseen phenomena, extant research strongly suggests that embodied actions can help learners better apprehend spatial transformations of those objects. Such transformations are varied and include predicting the outcome of a rotation or reflection as well as more complex dynamic spatiotemporal operations, such as the multiple transformations involved in simulating movement in a pulley system or gear train. In Chu and Kita’s (2008) work on gesture for promoting mental rotation, the researchers observed the highest performing participants to rotate their hands in a manner that suggested the participants were simulating the possible rotations of the stimuli with their hands. The use of embodied actions in this way has also been observed in the context of STEM learning. Kozhevnikov, Motes, & Hegarty (2007) showed that embodied actions are performed by STEM learners simulating kinematics in the domain of physics, while Stieff and colleagues showed that chemistry learners use their hands to simulate different molecular conformations that result from the internal rotation of atoms around bonds (Stieff et al., 2016) or the coupling of molecules in a reaction (Stieff, 2011).

The physiological limitations of human anatomy preclude using embodied actions to simulate all the various spatial operations learners are tasked with understanding in the context of STEM classrooms, but many spatial concepts can be mapped to embodied actions with high fidelity (Johnson-Glenberg, Birchfield, Tolentino, & Koziupa, 2014). For example, gestures can easily simulate different types of motion around geologic faults (Kastens, Agrawal, & Liben, 2008) or the movement of astronomical bodies in planetary systems (Padalkar & Ramadas, 2011). In fact, notoriously difficult abstract spatial concepts, such as quaternions in mathematics, torque in physics, or magnetic anisotropy in chemistry can readily be represented by embodied actions that involve moving only the hands and arms. We argue actions such as these should be the prime target for designers who wish to incorporate embodied action into STEM learning environments. High-fidelity actions present the dual benefit of permitting the learner to perform an action that would otherwise be simulated mentally and increasing the salience of the relationship between the embodied action and the spatial entities represented. Spatial concepts that require students to produce gesture or other actions that have a low degree of physical fidelity may present more challenges to the learner than possible benefits, as has been seen with learning environments that include low-fidelity visualizations in science classrooms (Collins, 1996). In the short term, embodied actions may serve as reinforcing representations (e.g., representational gestures) that can be reproduced as needed during problem solving. Then, with accrued experience of an embodied action, the need to overtly perform these actions should diminish as abstracted perception and action states become integrated into a learner’s mental representation.

### Design principle #3: Embodied learning environments should link innovative tools, such as visualizations or other simulations, to embodied actions through interface elements and input devices

As previously argued, the benefits of embodied action may result from the cueing of motor schemas or analog spatial representations during activity that are then linked to spatial concepts or disciplinary representations during learning. Given the increasing ubiquity of novel visualization technologies for supporting spatial learning in STEM disciplines and demonstrations of their efficacy, we argue that these technologies should work to link embodied actions with digital visualizations through input devices. We note that “gestural interfaces” that are designed to permit users to navigate through an interface without the use of a mouse or keyboard do not make use of the affordances of embodied action for learning as we have outlined here: opening browsers by tapping or swiping a screen to scroll through media do not recruit motor actions with any fidelity to spatial operations or link representational gestures to spatial entities. As such, designs such as these are not likely to support spatial learning. In contrast, designs that allow users to manipulate virtual objects as if they were tangible, present an interesting opportunity to support spatial learning by permitting users to perform embodied actions on visualizations of imperceptible or difficult to perceive phenomena. Leveraging both the embodied action and the visualization in tandem may yield far greater benefits than have been observed from either intervention alone.

In fact, preliminary models for coupling these technologies, and the resultant benefits, are already emerging. Barrett, Stull, Hsu, and Hegarty (2015) designed an application that allowed users to manipulate a virtual molecular model using a haptic input device. The device, a small plastic ovoid with embedded accelerometers, was linked to the application programming interface (API) in such a way that when handled it offered users the sense of physically interacting with the virtual model to interrogate spatial relationships between atoms and to observe the effects of rotating or twisting bonds on molecular configuration. In a series of studies, the authors documented that the technology was as effective as or better than handling physical models for helping users learn about stereochemistry and representational competence in chemistry. Moreover, the flexibility of the software environment offers new opportunities to scaffold spatial learning through constraining the degrees of freedom in a typical spatial operation so that it is easier to perceive the outcome of a transformation or by providing feedback on the outcome of a simulated action. Such work has been explored by Palmerius, Höst, & Schönborn (2012); they showed that gesture-based input devices that employ force touch sensors coupled to visualizations of biomolecules can help users easily learn how electrostatic interactions contribute to enzyme-substrate kinetics and molecular docking and recognition events.

Many concepts that require spatial thinking in STEM may readily be taught through the use of visualization technologies that leverage innovative input devices. Some of these entities we have addressed are too small to directly perceive (e.g., molecules), but there are other theoretical and conceptual entities in STEM that lack direct perceptual components on their own. Such concepts are also frequently invoked to reason about behaviors of physical systems (e.g., force in Newtonian mechanics, wave functions in quantum mechanics). Despite the inherent lack of perceptual features, it is common for scientists to construct external representations of these concepts (e.g., force vectors, mathematical models of electron density). These external representations then serve as tools with which to reason about inherently non-perceptual phenomena. Visualization, arguably, serves as one type of tool with which novel representations of complex concepts in STEM can be presented and coupled to embodied actions. It may be that the lack of regular perceptual access to various STEM phenomena is part of what makes them so challenging to learn. We would hypothesize that concepts that require spatial thinking, but lack direct perceptual character, may most effectively be taught via visualization technology aligned to embodied actions and yield greater gains for learners compared against traditional instruction.

## Toward applying embodied design principles: A pilot study

**Fig. 1:**
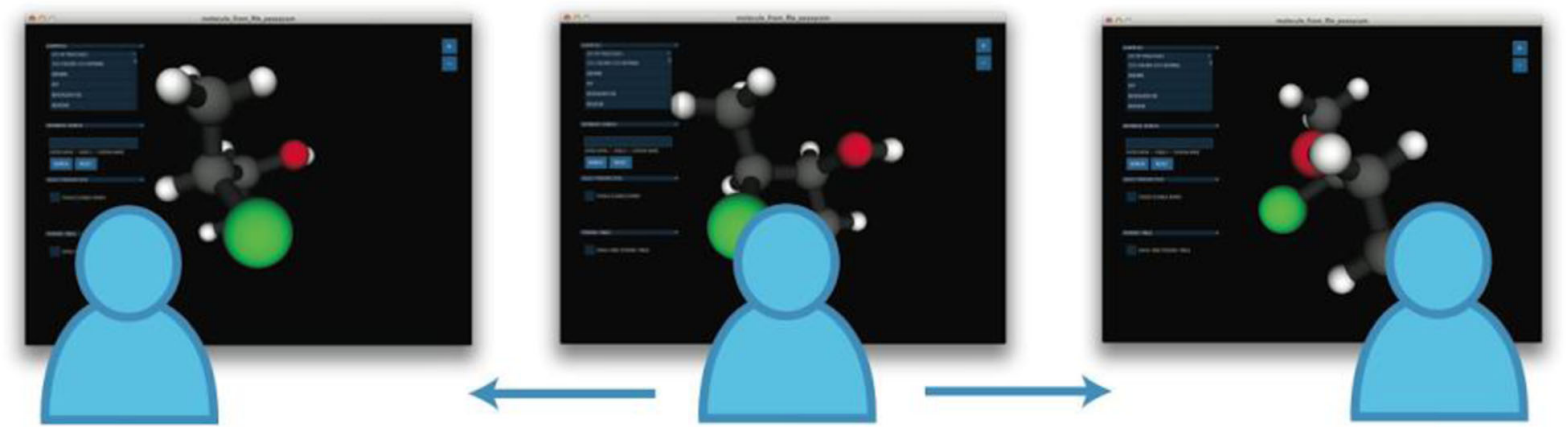
By shifting perspective from left to right, the molecular visualization interface changes to reorient the molecule with the learner’s assumed perspective

In our current research, we have attempted to explore the utility of these design principles for development and for research with an embodied interface that helps learners to apprehend spatial concepts and tools of representation in the discipline of organic chemistry. The embodied interface is a central component of a learning environment that merges perceptually rich, responsive computer technologies with scaffolded learning activities to teach students to apprehend and produce disciplinary diagrams that represent molecular structure. The technology (first reported in DeSutter & Stieff, 2014) belongs to a class of molecular visualization interfaces (e.g., Jmol, PyMOL, Avogadro) that use molecular structure data files to depict complex molecules as a visual representation of atoms (spheres) connected via single, double, or triple bonds (cylinders). Molecular visualization programs broadly speaking allow users to control the presentation of a molecule in virtual three-dimensional space via mouse movements. This software, though, differs from existing molecular visualizations by natively implementing computer vision algorithms that allow the learner to instead control the view of the visualization by physically changing their perspective relative to the screen. The face-tracking algorithm captures a student’s location relative to the computer screen and then dynamically updates the three-dimensional virtual scene as if these body movements actually occurred in the virtual environment. By leaning to the left or the right, the virtual scene shifts to show what would be seen if one could take the same vantage point on the rendered molecule in actual three-dimensional space. A depiction of how leaning from side to side controls the view of the interface can be seen in Fig. 1.

**Fig. 2:**
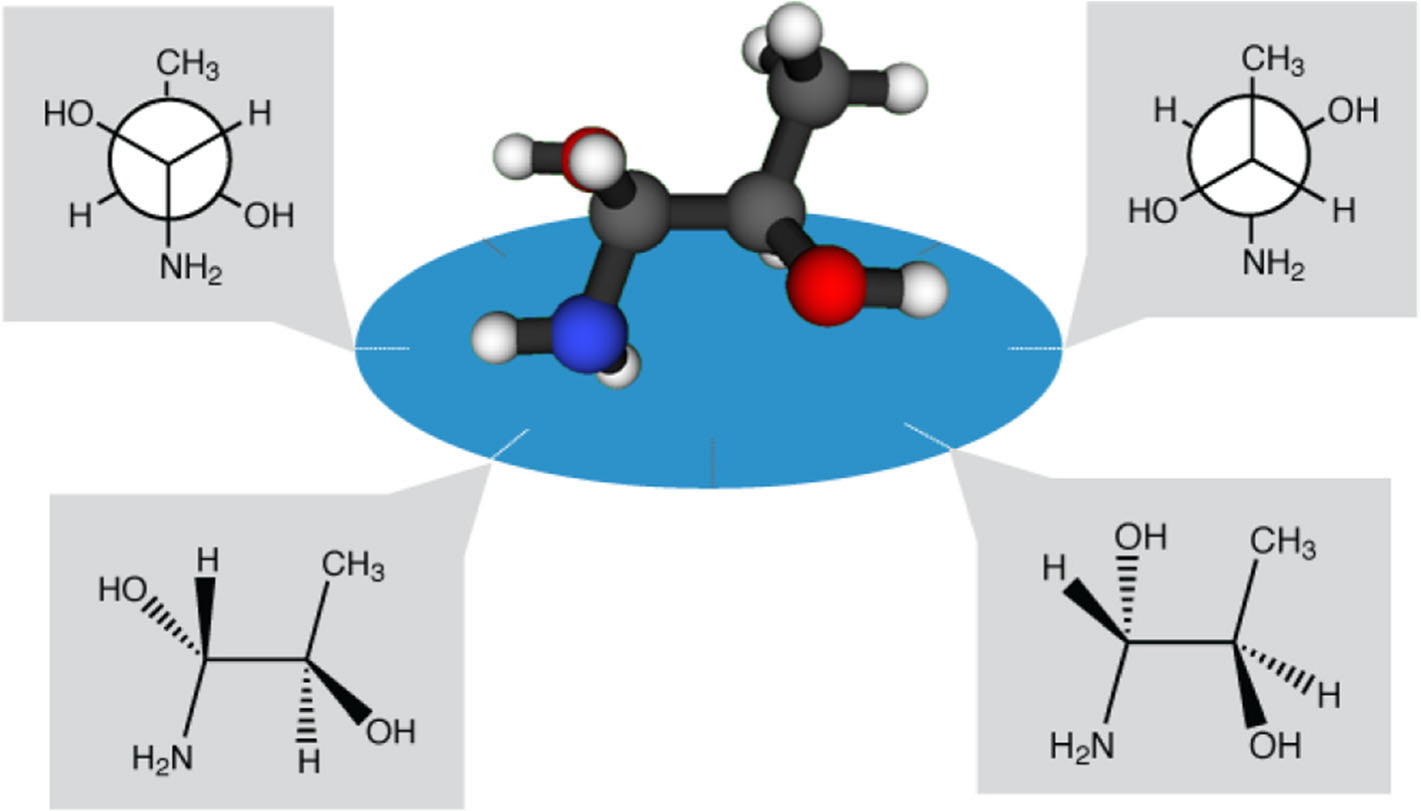
Equivalent structural diagrams of (*1R*,*2R*)-1-aminopropane-1,2-diol. A circle has been added beneath the structure of the molecule to show which vantage points coincide with the Newman (*top left*, *top right*) and Dash-Wedge (*bottom left*, *bottom right*) diagrams. Atoms are color coded to correspond to their identity: gray = carbon (*C*), white = hydrogen (*H*), red = oxygen (*O*), blue = nitrogen (*N*)

The embodied interface was designed with modularity in mind: it is intended to be used flexibly in learning environments that may differ in their learning objectives, but share a core commitment to investigating the role of embodiment in spatial thinking. In a recent pilot study, we tailored the design to investigate whether an embodied interface instantiating the aforementioned design principles could support students in understanding the complex spatial relationships in structural diagrams in organic chemistry. In the chemistry curriculum, students often struggle to interpret, relate, and transform representations as they reason about imperceptible phenomena (Kozma & Russell, 1997). Structural diagrams are a widely used communicating and reasoning tool in chemistry, as well as other STEM disciplines. Structural diagrams in organic chemistry are used to encode the spatial relationships of a molecule when it is viewed from particular angles. Two common diagrams that students are introduced to in their first semester are the Dash-Wedge and Newman projection diagrams (see Fig. 2). These diagrams can be understood as viewing a molecular structure from either a “side-on” (Dash-Wedge) or an “end-on” (Newman) perspective. Each diagram then deploys its own semiotic conventions to encode atomic identity, perspective in space, and relative spatial placement of atomic groups. Of importance, the same molecule can be viewed from multiple perspectives and multiple Dash-Wedge and Newman diagrams can be drawn to represent it. Figure 2 depicts how the molecule (*1R*,*2R*)-1-aminopropane-1,2-diol can be encoded in Dash-Wedge and Newman structural formalisms from multiple viewing perspectives. Note that a Newman diagram and a Dash-Wedge diagram of the same structure can be related to one another through the spatial transformation of a perspective shift.

**Fig. 3:**
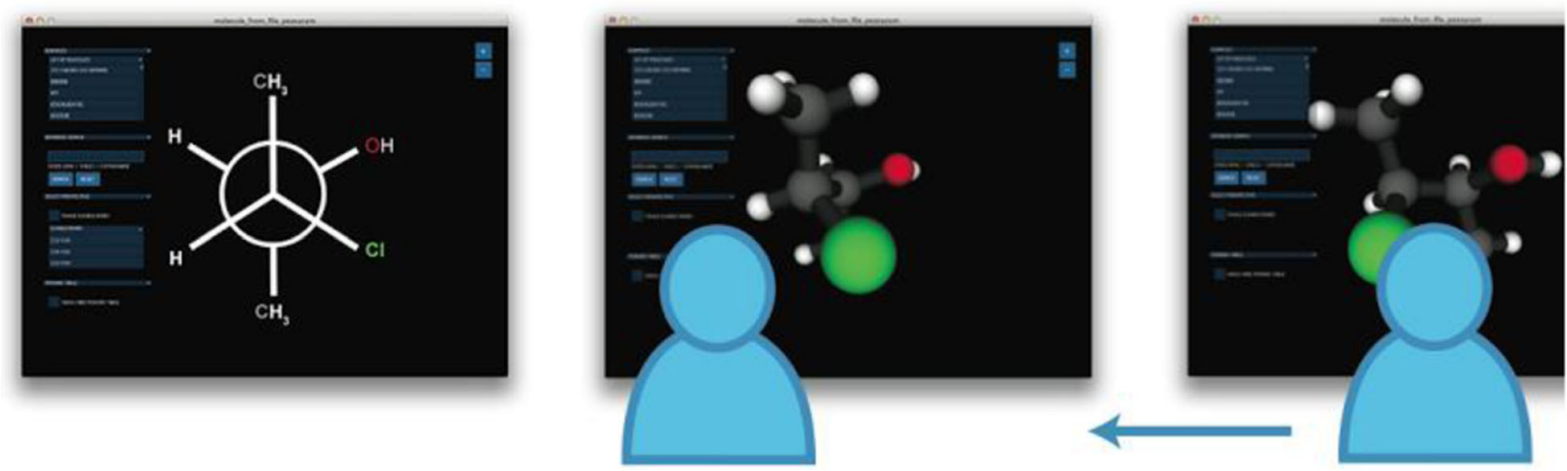
Structural diagrams (here the Newman diagram) are rendered as the participant aligns their perspective correctly. Students then copy this diagram down on paper

To investigate how this embodied interface supported students as they interpreted and related structural diagrams, we recruited naïve students (*n* = 6) to use the interface to explore how structural diagrams represent molecular structure and the spatial transformations that exist between them. The embodied interface was augmented to couple the face-tracking technology with changes in the visualization window. The interface was programmed to respond as a learner aligned their perspective to the vantage point encoded in either a Dash-Wedge or Newman diagram: the interface would swap out the three-dimensional ball and stick model with the correct structural diagram. Students were first taught to relate their movements to the different perspectives and representations through explicit scaffolds (Design principle #1): a think aloud protocol was designed that prompted students to use the interface to investigate three unique molecules and to draw at least one Dash-Wedge and one Newman diagram of each molecule on a provided piece of paper. An example of how the interface produced structural diagrams in response to physical perspective shifts is presented in Fig. 3.

Each participant was first guided to manipulate one molecule through specific perspectives and representations before freely exploring how structural diagrams could be drawn from the remaining two molecules. Participants were asked to perform discrete tasks and think aloud as they used the interface. For example, they were asked to explain how the virtual three-dimensional scene changed as they physically shifted their perspective, they were asked to demonstrate what vantage points coincided with each structural diagram, and they were prompted to explain how the physical movements they performed triggered different structural diagrams of the molecule to appear on screen. Moreover, when students reached the first example of a Dash-Wedge or Newman diagram, they were asked to explain how the spatial relationships in the structural diagram corresponded to the three-dimensional ball and stick rendering and then were directed to sketch the corresponding structural diagram on paper. The inclusion of scaffolding supports for students was critical given prior findings that the usefulness of any learning environment design can be undercut by not adequately considering the supports that students need to orient themselves properly to the learning activity.

A crucial feature of this design is the use of computer vision (OpenCV) to extend interaction beyond the keyboard and mouse paradigm. Students in the organic sequence are expected to know that a Newman diagram and a Dash-Wedge diagram of the same structure are related through a perspective shift. The face-tracking technology at the center of this embodied interface was embedded into the design so that learners could progress from having a notional idea of the spatial operation of *changing perspective* to having direct experience of moving between multiple structural diagrams. This design feature directly establishes a correspondence between the spatial operation of perspective shift and a high-fidelity motoric action (Design principle #2). From the theory of embodiment and proposed relationship to spatial thought, learners may struggle to mentally imagine shifting their perspective around a three-dimensional molecule in order to create structural diagrams without adequate prior perceptuomotor states to simulate. The embodied interface, thus, grounds perspective shifts in a tangible corporeal action that is at the disposal of the student.

**Fig. 4:**
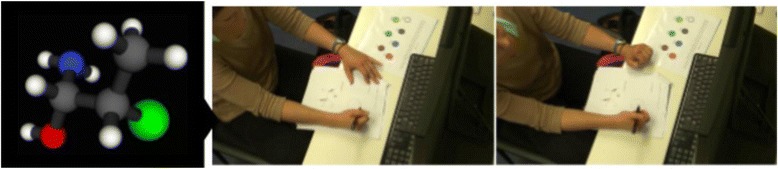
Students shifting their physical perspective as they work to draw a Newman perspective of (*1S*,*2S*)-1-amino-2-chloropropan-1-ol from a static image. Correctly drawing the Newman diagram requires students to mentally imagine shifting their perspective. After learning from the embodied interface, students reproduced the high-fidelity motoric action even though it no longer controlled the visual display

Following completion of the learning activity, participants were asked to draw a Dash-Wedge and a Newman diagram of a statically rendered molecule without the aid of the software interface. Of interest, we observed that some students continued to physically shift their perspective as they had done when using the interface. This occurred specifically when the participants attempted to draw the Newman projection, which required imagining a vantage point different from the perspective encoded in the image. Figure 4 shows a student drawing a Newman projection from a static image of (*1S*,*2S*)-1-amino-2-chloropropan-1-ol. Given that the image she sees is static, there should be no clear affordance of physically changing perspective. Such behavior suggests that instructing individuals with a high-fidelity embodied action influenced their immediate problem-solving strategy choice beyond the context of the embodied interface.

The visualization interface presented here uses only one of many forms of human–computer interaction that can link motoric action to the control of a molecular visualization interface for spatial thinking. Novel tools and technologies for improving spatial thinking should consider that the link between motoric action and technology interfaces has expanded profoundly since the invention of the mouse (Design principle #3). Various input devices are at the disposal of designers and have the potential to contribute to research around embodied actions for spatial thinking. In the case of our research, we wanted to know whether linking a motoric action that directly comports with how humans experience their environment might be useful in teaching about spatial perspective shifts. Other projects may have different research aims and we chose to leverage existing computer hardware enhanced by computer vision. Each learning environment embedding embodied actions to improve spatial thinking must ultimately justify the inclusion of any particular novel technology. These technologies must be justified in terms of their affordance for high-fidelity motoric action and how such action should improve knowledge of spatial concepts underlying disciplinary representation and processes of reasoning in the chosen STEM domain. The choices for designers are uncountable and span from enhancing traditional computer hardware with computer vision libraries, to novel input sensors like the LEAP motion controller that allows gestural control, to those that do not even require a traditional computer such as smartphone-driven virtual reality headsets.

Of importance, new investigations regarding embodied interfaces and spatial thinking in STEM must not only examine the efficacy of such interfaces for improving learning in real world contexts, they must also strive to reveal the precise causal mechanisms by which these interfaces support and improve spatial thinking. The interface we described here was built around all three design principles we enumerated to yield a design that could best support spatial thinking in chemistry in a preliminary investigation. Future investigations will need to compare the relative benefits of embodied actions that vary in the number and quality of scaffolds, as well as the extent to which those actions are analogous to imagined spatial operations and their integration with high quality visualizations. As above, the affordances of embodied actions for spatial thinking may result from their role as external representations that make spatial information more salient or their ability to cue relevant spatial motor schemas that help learners predict the outcome of a dynamic transformation. It remains unclear whether one or both of these mechanisms best explain the efficacy of embodied actions for promoting STEM learning, and alternative interfaces that make spatial information, both static and dynamic, more salient will need to be pitted against embodied interfaces such as we have described here.

## Conclusions

Training the next generation of STEM professionals will require a broad effort at all levels of reform to address the issues facing the modern student and designing new curricula and technology to support spatial thinking represent crucial targets in this effort (National Research Council, 2006). Various learning environments have emerged that aim to improve spatial thinking in STEM; however, despite some limited success, differing assumptions have been made about the nature of spatial thinking and the causal means to improve it. Rather than targeting domain-general competencies such as visuospatial ability, spatial thinking may need to be addressed within its disciplinary context of use, giving attention to the spatial concepts that underlie disciplinary concepts, how spatial concepts are instantiated in representational tools of that domain, and the spatial ways of thinking that contextualize the use of spatial concepts and representation in service of scientific inquiry. Embodied actions as we have argued may directly meet the needs of improving spatial thinking, given evidence that aspects of human knowledge and especially spatial knowledge and computation are grounded in perception and action simulation. Of importance, evidence suggests that embodied actions can influence spatial thinking by providing a body-based representation that foregrounds action in mental representation. Learning environments that merge embodied actions with representationally rich, domain-relevant visual displays, in particular, may serve to broadly improve real outcomes for STEM students.

An epistemic hurdle remains, though, for the research field. If the embodied/disembodied dichotomy has truly outlived its usefulness, then we are dealing with an expansive gray area where some aspects of knowledge and cognition are grounded in simulation, whereas others are not. What should be foregrounded in studies of embodiment is what we will accept as *evidence of embodiment* and, for those invested in learning, what serves as acceptable evidence that instruction via embodied action leads to a student embodying a concept that they had not embodied before. A number of studies have used an interference paradigm to argue for the existence of perceptuomotor simulation based on response time biases. These studies suggest that if a participant can be posed a task that requires simulation as part of semantic grounding, then asking them to move their bodies in ways contrary to what should be required of conceptual processing should give rise to detectable response time differences. For example, in the ACE, Glenberg and Kaschak (2002) empirically demonstrated that individuals comprehend action sentences slower when the motion the participant performs to press a button on a response box falls incongruent with the motion implied by the sentence. In fact, there is little doubt that these empirical findings are robust, as they have been replicated across multiple experimental contexts multiple times (Kaschak, Jones, Coyle, & Sell, 2009; cited in Chatterjee, 2010). However, “[what] does it mean to have quicker responses on the order of 10 to 100 milliseconds?” (Chatterjee, 2010, p. 85). It is not that someone’s thinking grinds to a halt when they are prevented from moving their hands or their physical action is mismatched to the one they are imagining during conceptual processing. Response time disparities are in fact predicted by theories of embodiment, but these metrics on their own cannot definitively explain *when* or *why* perceptual and motor systems are engaged during conceptual processing.

The notion that conceptual processing is accompanied by perceptual and motor activation is relatively uncontroversial. In fact, many neuroimaging studies have empirically demonstrated that the automatic observation of manipulable objects, comprehension of sentences that imply bodily action, and the observations of another’s actions all lead to clear demonstrable activation of the motor system (Mahon & Caramazza, 2008). Moreover, imagining an image activates 90% of the same neural substrates active during actual perception (Ganis et al., 2004; Kosslyn et al., 1997). What remain contested are the top-down models of conceptual processing that embodiment builds on. Whether perceptual and motor activation grounds concepts in the mind or is just an epiphenomenon of a more abstract conceptual knowledge cascading through these brain regions is not a settled matter. If the field is to continue arguing for the view that concepts are grounded in perception and action and that this manifests, quite literally, in the brain, then a mutually supporting collaboration must be established with neuroscientists to assure that our working assumptions about the embodied mind are not seriously undermined.

An open collaboration between educational and neuroscience research should prove to be mutually beneficial on this front. Neuroscientists are already posing questions about the nature of knowledge representation, memory, and cognitive processing and a number of these researchers agree that the evidence suggests a selectively embodied cognitive architecture (e.g., Chatterjee, 2010; Gallese & Lakoff, 2005; Meteyard *et al*., 2012). As natural scientists use instrumentation in the laboratory to hone their understanding and theory of physical systems, we should not rule out that the thesis of the embodied mind could also be honed from direct brain imaging studies. If educational researchers are to continue claiming that knowledge is embodied, then neuroscience provides direct empirical evidence of what is embodied and when. Learning environments can then scrutinize these models by deriving testable conjectures and embedding them in learning environments that explicitly put to the test how people learn. Such work is worthwhile because it also satisfies the need to provide feedback mechanisms for neuroscientists to examine how their top-down models of cognition fare in ecologically situated contexts.

Concepts are also a complex entity when we consider them at the level of classroom-based learning. It is *not* sufficient to claim that a disciplinary concept is embodied without some specificity around what we mean by *concept,* what that concept is composed of, and what precisely is being (or should be) embodied as an outcome of learning. One possibility may be to draw on framings in the literature around conceptual knowledge and its phenomenological basis (Smith, diSessa, & Roschelle, 1994; diSessa, 1988). This work argues that concepts are not stable monolithic entities, but rather are dynamic loose networks of interconnected knowledge elements that can rearrange in response to context and are composed of explanatory primitives (some that have a clear somatic basis) that can bear little resemblance to expert causal thinking. As we consider learning through embodied actions and what embodiment of conceptual knowledge looks like as an outcome of learning, we are likely to find a similar rich diversity beneath the surface. For example, rather than claiming that a student embodies the concept of “the water cycle,” maybe instead there are discrete composite features of a model conception of the water cycle that are grounded in simulation (e.g., the movement of water between reservoir and atmosphere, condensation, and evaporation) and others that are not (e.g., transfer of solar energy to kinetic energy).

While the research field settles on the evidentiary basis of claims of embodiment, arguably the best source of evidence at our disposal may be in both observing the visible manifestations of embodiment as well as experimentally manipulating the availability of embodied actions in an intervention. Gestures, as one well-studied example, have been argued to arise from simulations of motor action in the brain cascading to trigger the execution of visible motor programs, making it an observable manifestation of embodiment (Hostetter & Alibali, 2008). Cognitive processing and knowledge that evoke a strong perceptuomotor character should correlate to increased frequency of gesture use. In fact, it is a well-documented phenomenon that humans often gesture when talking about space (Alibali, 2005). This may render gesture as a useful proxy for detecting a learner’s underlying simulation and has potential to provide *in situ* evidence that embodiment is implicated in a student’s explanation or solution strategy.

Directly manipulating the availability of gesture may provide further evidence of embodiment. In the context of investigating the embodiment of spatial thought, manipulating the availability of gesture/embodied action on tasks that explicitly involve spatial reasoning should give rise to differences in group level and within-subject performance. The work of Chu and Kita (2011) stands out as an example of this methodology: the authors first manipulated between subjects whether participants were encouraged to gesture on a Shepard and Metzler-style block rotation task. They then gave a second assessment where they manipulated within-subject gesture availability. They found that individuals who were encouraged to gesture in the first task outperformed their peers on the second task, demonstrating that there was some durable effect of using their hands to reason about the complex spatial transformation beyond the immediate gestural act. Similar to a manipulation check, by altering the availability of embodied actions we can selectively observe, all other things being equal (*ceteris paribus*), whether embodied actions positively impact performance on the task at hand. Manipulating the availability of embodied actions provides a way to observe the connection between embodied actions and embodiment (i.e., offline simulation), especially when those taught to use their body on a task still outperform a control group in the absence of overt movement.

A decade on, the National Research Council’s (2006) call to action still resonates. Improving spatial thinking in STEM will require many learning environments, in many disciplines, over many iterations. Embodied actions, because they have the potential to ground abstract concepts of space and their forms in representation and reasoning in tangible body-based ways, may rather effectively allow learning environment designers to achieve their desired learning objectives. Our optimism is qualified by the need for a continued examination of the assumptions that support the embodiment thesis and that an open collaboration with neuroscientists may help the field converge on a shared model of an embodied cognitive architecture, its selective grounding of knowledge, and its responsiveness to change.
